# Modulation of Drug Resistance and Apoptotic Pathways Underlies the Enhanced Antitumor Effect of Ellagic Acid–Irinotecan Combination in Glioma

**DOI:** 10.3390/ijms27135814

**Published:** 2026-06-27

**Authors:** Burcu Biltekin, Abdurrahman Çetin

**Affiliations:** 1 Department of Histology and Embryology, Medical Faculty of Istanbul Atlas University, 34403 Istanbul, Turkey; 2 Department of Neurosurgery, Gazi Yaşargil Education and Research Hospital of Health Science University, 21010 Diyarbakır, Turkey; abdurrahmanctndeah@gmail.com

**Keywords:** glioma, ellagic acid, irinotecan, apoptosis, drug resistance

## Abstract

Gliomas account for over half of all primary malignant tumors of the central nervous system and remain associated with poor prognosis. Although irinotecan is an effective chemotherapeutic agent, its clinical utility is limited by systemic toxicity, prompting interest in phytochemicals such as ellagic acid (EA) as potential sensitizers. This study aimed to investigate whether EA enhances the antiproliferative and pro-apoptotic effects of irinotecan in C6 glioma cells. C6 glioma cells were treated with EA (100 µM), irinotecan (100 µM), or their combination for 24, 48, and 72 h. Cell proliferation was assessed by BrdU assay, p53 and caspase-3 protein expression by immunocytochemistry (H-SCORE), and multidrug resistance gene 1 (MDR1), MGMT, p53, and caspase-3 mRNA levels by RT-qPCR. EA significantly enhanced irinotecan-mediated suppression of proliferation at 24 h (*p* < 0.001), 48 h (*p* < 0.001), and 72 h (*p* < 0.001), with the combination producing the strongest inhibition across all time points. Immunocytochemical p53 expression increased significantly in all treatment groups at 24 h and 48 h (EA: *p* < 0.01; irinotecan: *p* < 0.01; EA + irinotecan: *p* < 0.01) and remained elevated at 72 h (*p* < 0.05). Caspase-3 immunoreactivity showed robust early activation at 24 h (Ir: *p* < 0.05; EA: *p* < 0.01), persisted at 48 h (*p* < 0.01), and remained significantly elevated in the EA group at 72 h (*p* < 0.001). At the mRNA level, irinotecan induced the highest p53 expression at 24 h (*p* < 0.001), with sustained elevation at 48 h and 72 h (*p* < 0.001 and *p* < 0.05, respectively). Caspase-3 mRNA peaked at 24 h only in the irinotecan group (*p* < 0.001). EA significantly increased MDR1 and MGMT transcription at 24 h and 48 h (*p* < 0.001), whereas the EA + irinotecan combination attenuated this increase and remained close to control levels at early time points. MGMT remained significantly elevated in EA and EA + irinotecan groups through 72 h (*p* < 0.001). EA cooperatively enhanced the antitumor activity of irinotecan primarily by enhancing proliferation inhibition and modulating drug-resistance gene expression, while maintaining, rather than further augmenting, apoptotic protein markers comparable to those induced by single-agent treatments. These findings support EA as a promising adjunct to irinotecan-based glioma therapy.

## 1. Introduction

Malignant gliomas are among the most aggressive primary brain tumors, originating from neural stem or progenitor cells that acquire tumor-driving genetic alterations. They account for approximately 26% of all intracranial tumors, with glioblastoma representing the most lethal subtype. Despite advances in therapeutic strategies, including radiotherapy and chemotherapy, patient prognosis has remained poor over the past decades [1,2]. One of the major challenges in glioma treatment is the limited delivery of therapeutic agents across the blood–brain barrier (BBB), as well as the rapid development of drug resistance.

Glioma cells develop resistance through multiple intrinsic mechanisms, including enhanced DNA repair capacity such as elevated MGMT expression, deficiencies in mismatch repair (MMR) pathways, dysregulation of tumor suppressor pathways including p53, and extensive metabolic reprogramming [3,4,5]. These mechanisms significantly reduce the effectiveness of conventional chemotherapeutic agents and highlight the need for novel combination strategies [6,7].

Irinotecan is a topoisomerase I inhibitor widely used in cancer therapy, including glioma. It induces DNA damage by interfering with DNA replication, leading to replication arrest and apoptosis. Importantly, irinotecan has been reported to cross the BBB and modulate multiple signaling pathways involved in tumor progression [8,9].

Natural compounds have gained increasing attention as adjuvant agents in cancer therapy due to their ability to modulate key cellular processes such as proliferation, apoptosis, and oxidative stress. Ellagic acid is a polyphenolic compound found in various fruits and vegetables and has been reported to exert anticancer effects through regulation of multiple molecular pathways, including apoptosis, inflammation, and oxidative stress [10,11,12,13,14]. Furthermore, ellagic acid has been shown to inhibit glioblastoma cell growth both in vitro and in vivo [11,14].

Combination therapies involving chemotherapeutic agents and natural compounds represent a promising strategy to overcome drug resistance and enhance therapeutic efficacy [12,13,14,15,16]. In our previous study, we demonstrated that the combination of ellagic acid and irinotecan enhanced antitumor activity in C6 glioma cells by reducing proliferation, modulating cadherin switching, and promoting antiangiogenic processes [14]. However, the molecular mechanisms underlying this combined effect remain insufficiently characterized.

Although previous studies from our group have explored the effects of ellagic acid in combination with chemotherapeutic agents [12,13,14], the present study differs in both scope and experimental focus. Specifically, this work evaluates the combined effects of ellagic acid and irinotecan in C6 glioma cells within a unified experimental design, integrating proliferation assays, immunocytochemical analysis, and gene expression profiling of key pathways involved in apoptosis, DNA repair, and multidrug resistance (MDR1, MGMT, p53, and caspase-3). This integrated approach allows a more comprehensive assessment of the molecular response to combination treatment compared to our previous reports. It should be emphasized that while the antiproliferative and molecular effects of ellagic acid alone on these markers have been partially addressed in our earlier reports, the effects of irinotecan administered alone, and in particular the molecular response to the EA–irinotecan combination, on MDR1, MGMT, p53, and caspase-3 expression have not been previously examined by our group or, to our knowledge, by others. The present study therefore extends beyond prior work by directly characterizing how irinotecan modulates these resistance- and apoptosis-related pathways, both independently and in combination with EA, which constitutes the principal novel contribution of this manuscript. We note that the experimental methodology and the three-time-point design (24, 48, and 72 h) were intentionally maintained from our previous work to allow direct comparison across treatment conditions; the novelty of the present study therefore lies in the treatment condition examined and the resulting molecular response, rather than in the experimental methodology itself.

Therefore, the present study aims to investigate the antiproliferative and molecular effects of irinotecan alone and in combination with ellagic acid in C6 glioma cells, with particular emphasis on drug resistance- and apoptosis-related markers, including MDR1, MGMT, p53, and caspase-3.

## 2. Results

### 2.1. Proliferation Index

**Figure 1:**
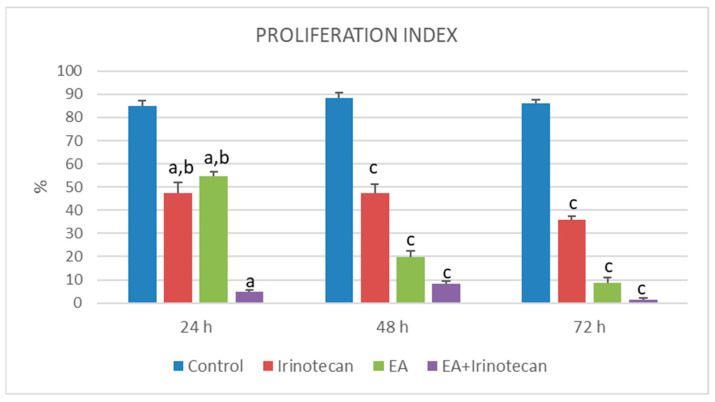
Proliferation index of C6 glioma cells following treatment with irinotecan, ellagic acid (EA), or their combination (EA + irinotecan) at 24, 48, and 72 h. Cell proliferation was assessed using the BrdU incorporation assay and expressed as a percentage relative to the control group. Data are presented as mean ± SEM from three independent experiments. Statistical annotations: a = *p* < 0.001 vs. control group; b = *p* < 0.001 vs. EA + irinotecan group; c = *p* < 0.001 vs. all groups.

The proliferation index of C6 glioma cells showed a time-dependent decrease in all treatment groups compared with the control (Figure 1). At 24 h, the control group maintained a high proliferation index (84.87 ± 2.25%). Both irinotecan (47.22 ± 1.90%) and EA (54.52 ± 4.69%) significantly reduced cell proliferation relative to the control group (*p* < 0.001), while the combined EA + irinotecan treatment produced the most pronounced suppression (5.01 ± 0.52%), showing a nearly complete inhibition of proliferation (*p* < 0.001 vs. all groups).

At 48 h, the antiproliferative effect became more distinct. The control group remained at 88.48 ± 2.37%, while irinotecan (47.25 ± 2.64%) showed moderate reduction, EA (19.75 ± 4.00%) showed a further decline, and the combination group (8.45 ± 1.00%) demonstrated a marked and statistically significant decrease compared to each individual treatment (*p* < 0.001) (Figure 1).

By 72 h, the control group maintained 86.10 ± 1.65%, and the lowest proliferation index was again observed in the EA + irinotecan group (1.52 ± 0.63%), which remained significantly reduced compared with the control, irinotecan alone (35.98 ± 2.24%), and EA alone (8.76 ± 1.51%) (*p* < 0.001 for all comparisons). Overall, the combination treatment consistently exhibited the strongest inhibitory effect across all time points, indicating an additive antiproliferative interaction between EA and irinotecan.

### 2.2. p53 Protein Expression

**Figure 2:**
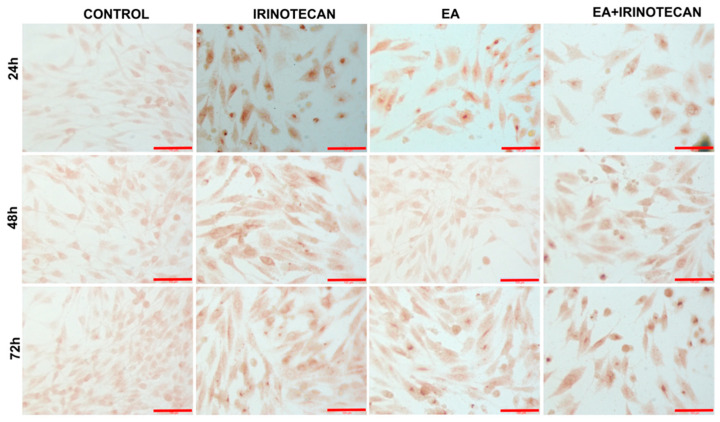
Microscopic images of immunocytochemical staining for p53 expression in C6 glioma cells following treatment with irinotecan, ellagic acid (EA), or their combination (EA + irinotecan) at 24, 48, and 72 h. ×400 magnification. Scale bar = 100 µm.

Immunocytochemical analysis demonstrated a marked increase in p53 immunoreactivity in all treatment groups compared with the control at each time point (Table 1; Figure 2). At 24 h, both EA and EA + irinotecan treatments showed significantly higher p53 staining compared with the control group (*p* < 0.01), while irinotecan alone produced a similar elevation. This trend persisted at 48 h, in which EA again exhibited significantly greater p53 expression relative to the corresponding control (*p* < 0.01), and the EA + irinotecan group showed a moderate but statistically significant increase (*p* < 0.05).

By 72 h, elevated p53 levels were maintained across all treatment groups, with EA and EA + irinotecan showing significantly higher immunoreactivity than the control (*p* < 0.05) (Table 1; Figure 2). Although the magnitude of expression varied slightly among treatments, the combined EA + irinotecan group maintained p53 immunoreactivity at levels comparable to those of individual treatments, without statistically significant differences from either monotherapy group across all time points.

### 2.3. Caspase-3 Protein Expression

**Figure 3:**
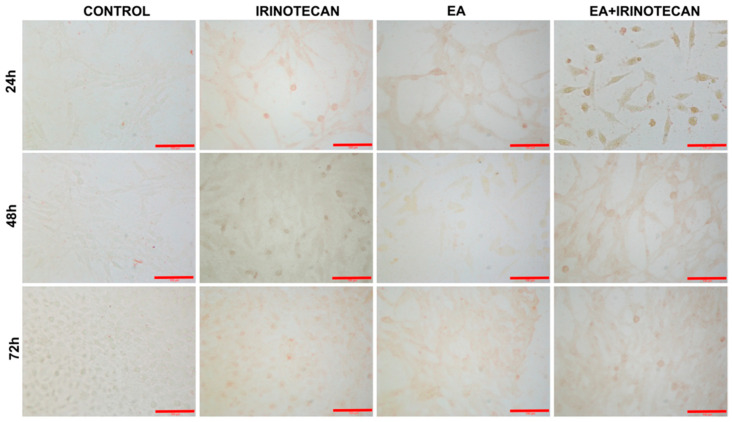
Microscopic images of immunocytochemical staining for caspase-3 expression in C6 glioma cells following treatment with irinotecan, ellagic acid (EA), or their combination (EA + irinotecan) at 24, 48, and 72 h. ×400 magnification. Scale bar = 100 µm.

Caspase-3 immunoreactivity demonstrated a pronounced early activation in response to all treatments, most notably at 24 h. At this time point, irinotecan and EA both produced significant elevations in caspase-3 expression compared with the control group (*p* < 0.05 and *p* < 0.01, respectively), while EA + irinotecan showed elevated caspase-3 immunoreactivity relative to control, though the combination did not exceed the response elicited by EA or irinotecan alone (Table 2; Figure 3).

At 48 h, the strong caspase-3 induction persisted, with both EA and EA + irinotecan treatments exhibiting significantly higher expression than the corresponding controls (*p* < 0.01). Irinotecan alone also maintained elevated caspase-3 levels at this time point (*p* < 0.01) (Table 2; Figure 3).

By 72 h, EA remained the only group with significantly enhanced caspase-3 expression compared with the control (*p* < 0.001), indicating a sustained pro-apoptotic effect. Irinotecan showed a moderate but significant increase (*p* < 0.05), whereas the combination group did not differ significantly from baseline (Table 2; Figure 3).

### 2.4. p53 mRNA Expression

**Figure 4:**
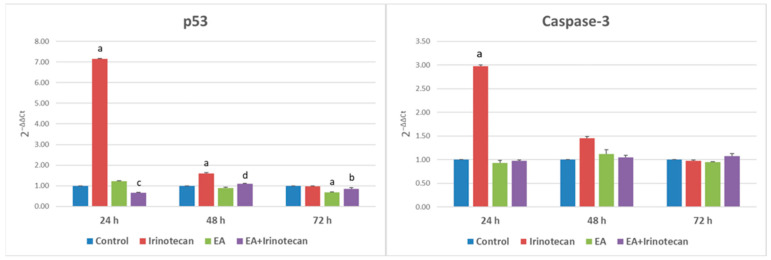
Relative mRNA expression levels of p53 and caspase-3 in C6 glioma cells following treatment with irinotecan, ellagic acid (EA), or their combination (EA + irinotecan) at 24, 48, and 72 h. Gene expression was quantified using the 2^−ΔΔCt^ method and normalized to GAPDH. Data are presented as mean ± SEM from three independent experiments. Statistical annotations: a = *p* < 0.001 vs. all groups; b = *p* < 0.05 vs. irinotecan; c = *p* < 0.05 vs. all groups; d = *p* < 0.05 vs. ellagic acid.

p53 mRNA levels showed distinct, time-dependent alterations across treatment groups (Figure 4). At 24 h, irinotecan produced a pronounced increase in p53 expression (7.154 ± 0.029-fold relative to control), reaching the highest level among all groups (*p* < 0.001). In contrast, EA (1.214 ± 0.024-fold) and EA + irinotecan (0.658 ± 0.017-fold) showed significantly lower p53 expression relative to irinotecan (*p* < 0.05), and EA + irinotecan even displayed a mild reduction compared to control (*p* < 0.05).

At 48 h, irinotecan again induced a strong elevation in p53 (1.603 ± 0.049-fold; *p* < 0.001 vs. all groups), while EA + irinotecan generated a modest but significant increase (1.094 ± 0.031-fold) relative to EA alone (0.880 ± 0.040-fold; *p* < 0.05). EA-treated cells maintained lower p53 expression than all other groups (*p* < 0.05). By 72 h, irinotecan (0.966 ± 0.021-fold) and EA + irinotecan (0.850 ± 0.059-fold) maintained significantly higher p53 levels than EA alone (0.681 ± 0.030-fold; *p* < 0.05), while EA treatment remained comparable to or slightly lower than baseline levels (Figure 4).

### 2.5. Caspase-3 mRNA Expression

Caspase-3 mRNA showed a markedly elevated expression pattern only in response to irinotecan at 24 h (2.977 ± 0.050-fold), where a significant increase was observed compared with all other treatment groups (*p* < 0.001). EA (0.933 ± 0.028-fold) and EA + irinotecan (0.972 ± 0.016-fold) treatments did not produce a similar increase at this early point.

At 48 and 72 h, caspase-3 expression levels converged across all groups: irinotecan (1.451 ± 0.087-fold at 48 h; 0.978 ± 0.014-fold at 72 h), EA (1.120 ± 0.042-fold at 48 h; 0.947 ± 0.018-fold at 72 h), and EA + irinotecan (1.045 ± 0.045-fold at 48 h; 1.070 ± 0.055-fold at 72 h), with no statistically significant differences relative to control at either time point.

### 2.6. MDR1 mRNA Expression

**Figure 5:**
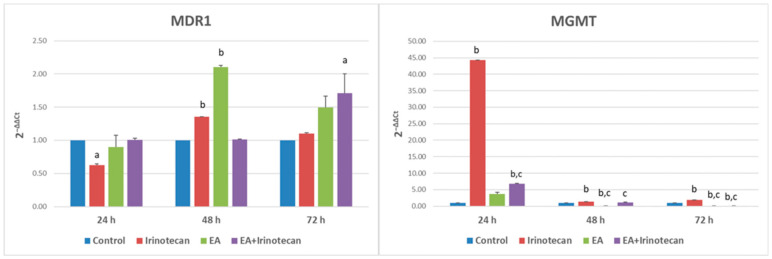
Relative mRNA expression levels of MDR1 and MGMT in C6 glioma cells following treatment with irinotecan, ellagic acid (EA), or their combination (EA + irinotecan) at 24, 48, and 72 h. Gene expression was quantified using the 2^−ΔΔCt^ method and normalized to GAPDH. Data are presented as the mean ± SEM of three independent experiments. Statistical annotations: a = *p* < 0.05 vs. control; b = *p* < 0.001 vs. control; c = *p* < 0.001 vs. irinotecan group.

MDR1 showed time-dependent expression changes following treatment. At 24 h, irinotecan slightly decreased MDR1 levels (0.627 ± 0.177-fold) compared with the control, whereas EA caused a modest but significant upregulation (0.896 ± 0.020-fold; *p* < 0.05). The combination group (1.004 ± 0.024-fold) remained comparable to the control at this early point (Figure 5).

At 48 h, EA induced the highest elevation in MDR1 expression (2.104 ± 0.004-fold; *p* < 0.001 vs. control), followed by irinotecan (1.352 ± 0.026-fold; *p* < 0.05 vs. control). However, the combined EA + irinotecan treatment (1.012 ± 0.005-fold) did not potentiate this effect and remained close to baseline levels. By 72 h, MDR1 expression decreased toward near-control values in the irinotecan group (1.101 ± 0.170-fold) but remained significantly elevated in both the EA (1.497 ± 0.012-fold) and EA + irinotecan (1.711 ± 0.290-fold) groups (*p* < 0.05 vs. control) (Figure 5).

### 2.7. MGMT mRNA Expression

MGMT showed highly dynamic responses to the treatments. At 24 h, EA markedly upregulated MGMT expression (3.681 ± 0.044-fold), while the EA + irinotecan combination also showed a significant rise (6.820 ± 0.082-fold; *p* < 0.001 vs. control). Strikingly, irinotecan alone induced a dramatic elevation (44.289 ± 0.534-fold; *p* < 0.001 vs. control and vs. EA groups).

At 48 h, MGMT expression levels decreased substantially. Irinotecan (1.384 ± 0.022-fold) and EA + irinotecan (1.142 ± 0.052-fold) remained significantly higher than controls (*p* < 0.001), whereas EA showed a marked decrease below baseline (0.052 ± 0.001-fold). By 72 h, irinotecan (1.911 ± 0.034-fold) and EA + irinotecan (0.071 ± 0.002-fold) maintained altered MGMT expression relative to control (*p* < 0.001), while EA (0.099 ± 0.002-fold) similarly remained suppressed below control levels (Figure 5).

## 3. Discussion

Malignant glioma displays marked heterogeneity due to a complex tumor microenvironment, tumor cells, tumor stem cells, diverse infiltrated immune cells and acquired strategies to evade treatment [17]. Moreover, the brain resides in a well-protected region isolated by the BBB, which represents one of the major challenges in glioma for drug administration. Given that, treatment of glioma requires more precise and individualized approaches to reduce mortality rates [3,4]. The conventional antitumor agent irinotecan, a topoisomerase I inhibitor, has been applied to various cancer types, including glioma and is known to cross the BBB [8,9]. SN-38 is the pharmacologically active metabolite of irinotecan and functions as the cytotoxic component. Irinotecan acts as a prodrug that undergoes hepatic biotransformation to yield SN-38 as its principal active form. Nevertheless, a significant fraction of SN-38 undergoes additional metabolism to form the inactive conjugate SN-38 glucuronide (SN-38G) via the UDP-glucuronosyltransferase UGT1A1, a pathway implicated in the gastrointestinal toxicity observed during irinotecan treatment. Employing a cooperative drug combination offers the additional benefit of enabling dose reduction, thereby minimizing the likelihood of adverse side effects [18,19]. In light of this knowledge, we combined irinotecan with EA, a natural phenol that serves as an anticarcinogenic, antioxidant, and antifibrotic agent [11,12,13,14]. Previously, we revealed that the combination of irinotecan with EA elevated antitumor activity and the cooperative effect of this combination reduced cell proliferation of C6 glioma by inhibiting the cadherin switch and promoting the antiangiogenic processes; however, further studies are required [14]. That prior report, however, did not examine drug resistance- or apoptosis-related gene and protein expression, nor did it address the molecular response to irinotecan administered alone. In this study, we co-treated C6 glioma cells with irinotecan and EA and scrutinized the molecular pathways involved in MDR, DNA repair and apoptosis. The combined treatment robustly suppressed cell proliferation compared with the control and the irinotecan-alone or EA-alone groups. These data indicate EA has the potential to additively enhance the antiproliferative effect of irinotecan. In the context of drug resistance, which is ubiquitous in glioma, and DNA repair mechanisms, MDR1 and MGMT were investigated at the mRNA level. Although EA remarkably upregulated MDR1 expression, irinotecan appeared to attenuate this overexpression when administered together with EA, suggesting that irinotecan may counterbalance EA-induced chemoresistance signals at early time points (24 and 48 h). The paradoxical upregulation of MDR1 by EA, as well as the overall temporal pattern of MDR1 expression, is consistent with our previous reports [12,14]. The novel observation in the present study is that irinotecan co-administration attenuates this EA-induced MDR1 upregulation, an interaction that was not addressed in our earlier work.

Methylation of the O^6^-methylguanine-DNA methyltransferase (MGMT) promoter is recognized as a prognostic and potentially predictive biomarker in glioblastoma; however, its utility in guiding therapeutic decision-making for patients remains a matter of debate. Methylation status in the MGMT promoter and its activity can compromise the sufficiency of alkylating drugs via drug resistance [20,21]. Our findings indicate that EA upregulated MGMT expression, whereas co-administration with irinotecan attenuated this induction, suggesting that irinotecan may partially counteract EA-driven DNA repair pathway activation. These results reflect a complex regulatory interplay in which EA sensitizes glioma cells to irinotecan despite increasing the transcription of resistance-associated genes, MDR1 and MGMT. This apparent paradox may be reconciled by considering the substrate specificity of P-gp and the mechanistic irrelevance of MGMT to topoisomerase I inhibitor-based cytotoxicity. Regarding MDR1, it should be noted that P-gp-mediated efflux preferentially affects hydrophobic substrates and, while SN-38, the active metabolite of irinotecan, has been reported as a P-gp substrate, irinotecan itself demonstrates relatively low affinity for P-gp-mediated transport. This may explain why MDR1 transcriptional upregulation did not translate into functional resistance to irinotecan in the proliferation assay. Regarding MGMT, this enzyme specifically repairs O6-alkylguanine adducts generated by alkylating agents such as temozolomide and dacarbazine. Since irinotecan exerts its cytotoxic effect through topoisomerase I inhibition rather than DNA alkylation, MGMT upregulation is not expected to directly confer resistance to irinotecan. Furthermore, the attenuation of both MDR1 and MGMT upregulation observed in the EA + irinotecan combination group relative to EA alone suggests that irinotecan may partially suppress EA-driven transcriptional activation of resistance pathways, thereby contributing to the net sensitizing effect observed at the functional level.

Moreover, the apoptotic profile supports this sensitization effect. To investigate apoptosis-related gene expression, p53 and caspase-3 genes were selected as key molecular targets. The *p53* gene is a well-known tumor suppressor; furthermore, its expression can induce a wide range of cellular effector mechanisms, including cell-cycle arrest, induction of senescence, regulation of multiple DNA damage–repair pathways, metabolic reprogramming, and apoptosis [22]. At the mRNA level, irinotecan produced the most robust p53 induction at 24 h, whereas at the protein level, EA exhibited comparable immunoreactivity. It should be noted that p53 is primarily regulated at the post-translational level through MDM2-mediated proteasomal degradation; therefore, increased p53 immunoreactivity observed by immunocytochemistry may reflect protein stabilization rather than transcriptional upregulation, and the two measurements are not directly interchangeable. The discordance between p53 mRNA and protein levels, particularly at 24 h, where irinotecan induced the highest transcriptional response yet did not produce proportionally greater immunoreactivity, suggests that post-translational regulatory mechanisms, including MDM2-mediated degradation and phosphorylation-dependent stabilization, may play a dominant role in determining the net p53 protein accumulation in this model.

Caspase-3 immunoreactivity also indicated a time-dependent pattern that complements the p53 response. EA sustained caspase-3 protein immunoreactivity through 72 h, whereas the combination group did not demonstrate statistically significant elevation relative to control at this time point, indicating that EA may prolong apoptotic signaling beyond the initial phase induced by irinotecan. The combined treatment resulted in augmented early caspase-3 activation yet did not surpass the response induced by EA alone at later intervals, suggesting that the cooperative effect between EA and irinotecan is likely more substantial in inhibiting proliferation than in driving late-phase caspase activation. Taken together, these immunocytochemical findings suggest that EA may contribute to apoptotic signaling while maintaining the cytotoxic effects of irinotecan without suppressing key apoptotic markers such as p53 and caspase-3.

Although the combination treatment did not produce statistically superior p53 or caspase-3 immunoreactivity compared with either monotherapy as assessed by immunocytochemistry, this finding should be interpreted in context. Both EA and irinotecan individually induced robust apoptotic marker expression, suggesting that a ceiling effect may have been reached at the protein level. The primary advantage of the combination was instead manifested in two distinct dimensions: a significantly greater suppression of cell proliferation compared with either agent alone, and a concurrent attenuation of EA-induced MDR1 and MGMT upregulation when irinotecan was co-administered. This multi-dimensional regulatory profile, rather than additive apoptotic protein induction, underlies the enhanced antitumor efficacy of the EA–irinotecan combination. It should be acknowledged that the apoptotic mechanisms proposed herein are largely inferred from semi-quantitative immunocytochemical and transcriptional data and would require validation through functional apoptosis assays and quantitative protein analyses in future studies.

An important observation in the present study is the discordance between mRNA and protein levels for both p53 and caspase-3. At the transcriptional level, irinotecan produced the most robust p53 and caspase-3 mRNA induction, particularly at 24 h. However, at the protein level as assessed by immunocytochemistry, EA-treated cells exhibited comparable or even higher p53 and caspase-3 immunoreactivity than irinotecan-treated cells. Such mRNA–protein discordance is well-documented in the literature and may arise from several mechanisms, including differences in mRNA stability and translational efficiency, post-translational modifications such as phosphorylation and ubiquitination that regulate protein half-life, and the activity of microRNAs or RNA-binding proteins that modulate translational output. In the context of p53 specifically, protein stabilization via MDM2 inhibition represents a major post-translational mechanism that operates independently of transcriptional changes. These findings underscore the importance of evaluating both transcriptional and translational outputs when characterizing apoptotic responses to combinational treatments.

This study has limitations. First, in vitro models cannot fully recapitulate the complexity of the brain tumor microenvironment, including BBB dynamics, cellular heterogeneity, and immune interactions. Second, the use of a single rat glioma cell line (C6) represents an important limitation, as C6 cells may not fully reflect the genetic, epigenetic, and phenotypic diversity of human gliomas. In particular, C6 cells harbor a wild-type p53 background and exhibit distinct molecular characteristics compared with human glioblastoma cell lines such as U87MG, U251, or T98G. Validation of the EA–irinotecan combination in human glioma models, including both established cell lines and patient-derived glioblastoma stem cells, will be essential to determine the translational relevance of these findings.

Third, although the present study demonstrated significant antiproliferative and pro-apoptotic effects of the EA–irinotecan combination, the underlying molecular mechanisms were not comprehensively investigated. The mechanistic interpretations proposed herein are primarily based on BrdU proliferation analysis, immunocytochemical evaluation, and RT-qPCR assessment of selected apoptosis- and drug resistance-related markers. Therefore, the observed alterations in MDR1, MGMT, p53, and caspase-3 should be considered preliminary mechanistic evidence rather than a complete elucidation of the molecular pathways involved. The protein-level quantification in this study was performed exclusively by immunocytochemistry using H-SCORE analysis, which, while validated and widely used, is inherently semi-quantitative. The absence of Western blot or ELISA-based protein quantification for p53 and caspase-3 represents a limitation. Similarly, apoptosis was inferred from molecular markers rather than directly quantified through functional assays such as Annexin V/propidium iodide flow cytometry or terminal deoxynucleotidyl transferase dUTP nick end labeling (TUNEL). These methodological constraints necessitate caution in interpreting the proposed apoptotic mechanisms as conclusive, and future studies should incorporate orthogonal protein quantification and functional apoptosis assays to substantiate these findings. Additionally, direct measurement of enzymatic caspase-3 activity using functional assays was not performed and represents a further limitation that should be addressed in future studies. Fourth, formal synergy analysis using established pharmacological models such as the Chou–Talalay combination index method or Bliss independence model was not performed. Consequently, the enhanced antitumor effect observed with the EA–irinotecan combination cannot be classified as synergistic, additive, or antagonistic with statistical certainty. Future studies employing dose-matrix experimental designs will be necessary to formally characterize the nature of this pharmacological interaction. Fifth, while MDR1 and MGMT mRNA upregulation was observed following EA treatment, functional drug efflux activity and DNA repair capacity were not directly measured. Future studies incorporating P-gp efflux assays and MGMT enzymatic activity measurements would be necessary to determine whether the transcriptional changes observed translate into functional resistance. Sixth, the use of a single fixed concentration for both EA and irinotecan, rather than a range of doses, represents an additional limitation of this study. Dose–response experiments and IC_50_ determinations for each agent individually and in combination were not performed, which precluded formal interaction analysis and limits the interpretation of the combined effect. Future studies should employ a matrix of drug concentrations to enable dose–response characterization, IC_50_-based concentration selection, and formal synergy assessment using established pharmacological models. Finally, validation of these findings in vivo glioma models is essential to assess translational applicability and therapeutic feasibility.

## 4. Materials and Methods

### 4.1. Cell Culture

C6 glioma cells (ATCC, Manassas, VA, USA) were maintained in Dulbecco’s Modified Eagle Medium (DMEM) supplemented with 10% fetal bovine serum, 100 U/mL penicillin, and 100 μg/mL streptomycin. Cultures were incubated at 37 °C in a humidified atmosphere containing 5% CO_2_. Cells were routinely subcultured every three days using trypsin.

For treatment experiments, ellagic acid (EA; Sigma-Aldrich, E2250-10G, St. Louis, MO, USA) was applied at a final concentration of 100 μM. Irinotecan hydrochloride (Onco-Tain™, Hospira, Lake Forest, IL, USA; 20 mg/mL, 100 mg/5 mL) was used at 100 µM.

The concentration of 100 µM for both EA and irinotecan was selected based on previously published in vitro studies from our group and others demonstrating antiproliferative and pro-apoptotic effects in glioma cell lines at this range [12,13,14]. These concentrations were not derived from formal IC_50_ determinations in the C6 cell line within the present study, and we acknowledge this as a limitation. Both compounds were applied at equimolar concentrations to allow direct comparison of their individual and combined effects.

Cells were allocated into four experimental groups for each incubation period (24, 48, and 72 h): control, irinotecan alone, EA alone, and combined irinotecan + EA. All experiments were independently repeated three times (n = 3) on separate occasions using distinct cell passages. Within each independent experiment, all treatment groups were evaluated in triplicate. Data are presented as mean ± SEM of three independent biological replicates unless otherwise stated.

### 4.2. BrdU Proliferation Assay

Cell proliferation was assessed using a BrdU incorporation method adapted from established protocols [23]. All assays were conducted in technical triplicate and repeated in three independent experiments (n = 3). 5-Bromo-2′-deoxyuridine (BrdU; SC-32323, Santa Cruz Biotechnology, Santa Cruz, CA, USA) and Histostain-Plus detection reagents (SensiTek ScyTek Laboratories, Logan, UT, USA) were used.

Following incubation, cells were treated with a mouse monoclonal anti-BrdU antibody (Bu20A, SC-20045; Santa Cruz Biotechnology) at 1:200 dilution overnight. AEC chromogen (SensiTek ScyTek Laboratories) was applied for signal development.

BrdU-positive nuclei were evaluated by two independent observers, and the proliferation index was calculated by counting at least 3000 cells per slide (BrdU-positive cells/total counted cells).

### 4.3. Immunocytochemical Assessment of p53 and Caspase-3 Protein Expression

C6 cells seeded on coverslips were treated with EA, irinotecan, or their combination and collected after 24, 48, or 72 h. Cells were fixed using cold methanol for 5 min. Immunostaining for p53 and active caspase-3 was performed using an indirect streptavidin-HRP technique (SensiTek ScyTek Laboratories, Logan, UT, USA).

Caspase-3 immunostaining was performed to assess protein-level apoptotic activation, using anti-caspase-3 antibody (AB3623; Millipore, Darmstadt, Germany) applied overnight at 4 °C. Similarly, p53 immunostaining was performed to assess tumor suppressor protein expression, using anti-p53 antibody (orb136435; Biorbyt, Hayward, CA, USA) applied overnight at 4 °C following manufacturer-recommended dilutions. The antigen–antibody complexes were visualized using AEC substrate.

All immunocytochemical experiments were performed in triplicate across three independent experiments (n = 3). Staining intensity was semi-quantitatively assessed using the H-SCORE method, as described previously [23].

### 4.4. Gene Expression Analysis of Apoptotic and Drug Resistance Markers

Total RNA was isolated using the Total RNA Purification Kit (Jena Bioscience, Jena, Germany). cDNA synthesis was performed using the SCRIPT cDNA Synthesis Kit (Jena Bioscience). Quantitative real-time PCR (RT-qPCR) was carried out with qPCR GreenMaster UNG (Jena Bioscience) on a CFX96 Touch System (Bio-Rad, Hercules, CA, USA). Primers specific for MDR1, F: 5′-CAGTTCATTCGCTCCTGACTAC-3′ and R: 5′-CGTGCTGTAGCTGTCAATCT-3′; for MGMT, F: 5′-GAAGCCTATTTCCACGAACCT-3′ and R: 5′-CACCTGTCTGGTGAATGAATCT-3′; for p53, F: 5′-ACATGACTGAGGTCGTGAGA-3′ and R: 5′-GATTTCCTTCCACCCGGATAAG-3′; for caspase-3, F: 5′-CTGACTGGAAAGCCGAAACT-3′ and R: 5′-GTTCCACTGTCTGTCTCAATACC-3′; for GAPDH (housekeeping gene), F: 5′-GCAAGGATACTGAGAGCAAGAG-3′ and R: 5′-GGATGGAATTGTGAGGGAGATG-3′. MDR1 mRNA expression was quantified to evaluate transcriptional regulation of multidrug resistance in response to treatments. MGMT mRNA expression was quantified to evaluate transcriptional regulation of DNA repair capacity and chemoresistance in response to treatments. p53 mRNA expression was quantified to evaluate transcriptional regulation of tumor suppressor signaling in response to treatments. Caspase-3 mRNA expression was quantified to evaluate transcriptional regulation of apoptotic signaling in response to treatments.

All RT-qPCR experiments were performed in technical triplicate and repeated across three independent biological replicates (n = 3). Relative mRNA expression levels were calculated using the ΔΔCt method, normalized to GAPDH, and expressed as mean ± SEM.

### 4.5. Statistical Analysis

All semi-quantitative and quantitative outcomes were analyzed using GraphPad Instat v3.06 (GraphPad Inc., San Diego, CA, USA). Prior to parametric testing, data distribution was assessed for normality. Results are presented as mean ± SEM from three independent experiments. Differences among treatment groups at each time point were assessed using one-way ANOVA followed by the Tukey–Kramer post hoc test for multiple comparisons. For the BrdU proliferation assay and RT-qPCR data, statistical comparisons were performed separately for each time point (24, 48, and 72 h). For immunocytochemical H-SCORE data, between-group comparisons were performed at each time point independently. No additional correction for multiple testing across outcome measures was applied, as each biomarker was analyzed as an independent, pre-specified biological endpoint. A *p*-value < 0.05 was considered statistically significant, *p* < 0.01 moderately significant, and *p* < 0.001 highly significant.

## 5. Conclusions

In summary, our findings indicate that ellagic acid significantly enhances the antitumor efficacy of irinotecan in C6 glioma cells by simultaneously modulating proliferation, apoptosis, and drug-resistance pathways. EA not only potentiates irinotecan-induced cytotoxicity but also sustains apoptotic marker expression and partially mitigates chemoresistance-associated gene upregulation. This multi-dimensional regulatory interplay suggests that EA may serve as a promising adjunct to irinotecan-based regimens, offering an effective strategy to improve therapeutic outcomes in glioma. Future studies incorporating in vivo models and BBB-penetration assessments will be critical to determining the clinical relevance and translational potential of EA–irinotecan combination therapy.

## Figures and Tables

**Table 1:** Immunocytochemical findings of p53 expression.

Groups	24 h	48 h	72 h	*p* Value
Control	7.80 ± 3.8	5.40 ± 3.2	6.00 ± 2.6	0.6650
Irinotecan	32.60 ± 5.1	27.00 ± 3.9	29.00 ± 3.6	0.3257
EA	36.40 ± 6.4 ^a^	33.40 ± 4.2 ^a^	34.00 ± 4.8 ^b^	0.7709
EA + Irinotecan	32.40 ± 4.6	31.80 ± 4.4 ^b^	33.80 ± 4.1 ^b^	0.6676
*p* value	0.0089	0.0042	0.0047	

^a^ *p* < 0.01 vs. corresponding control group, ^b^ *p* < 0.05 vs. corresponding control group.

**Table 2:** Immunocytochemical findings of caspase-3 expression.

Groups	24 h	48 h	72 h	*p* Value
Control	5.60 ± 1.5	6.00 ± 1.6	6.80 ± 1.6	0.4653
Irinotecan	33.80 ± 5.9 ^a^	26.40 ± 3.2	26.80 ± 3.1	0.0486
EA	36.20 ± 3.4 ^b^	35.40 ± 1.7 ^b^	36.00 ± 3.2 ^c^	0.9763
EA + Irinotecan	28.80 ± 3.3	29.40 ± 5.9	30.40 ± 0.9	0.4675
*p* value	0.0025	0.0021	0.0006	

^a^ = *p* < 0.05 vs. corresponding control group, ^b^ = *p* < 0.01 vs. corresponding control group, ^c^ = *p* < 0.001 vs. corresponding control group.

## Data Availability

The original contributions presented in this study are included in the article. Further inquiries can be directed to the corresponding author.
